# The Regulation of Anthocyanin Synthesis in the Wheat Pericarp

**DOI:** 10.3390/molecules191220266

**Published:** 2014-12-04

**Authors:** Olesya Y. Shoeva, Elena I. Gordeeva, Elena K. Khlestkina

**Affiliations:** 1 Institute of Cytology and Genetics, Siberian Branch, Russian Academy of Sciences, Lavrentjeva ave. 10, Novosibirsk 630090, Russia; E-Mails: elgordeeva@bionet.nsc.ru (E.I.G.); khlest@bionet.nsc.ru (E.K.K.); 2 Novosibirsk State University, Pirogova St. 2, Novosibirsk 630090, Russia

**Keywords:** *Triticum aestivum*, purple grain pericarp, candidate gene, regulatory gene, nucleotide sequence, transcription

## Abstract

Bread wheat producing grain in which the pericarp is purple is considered to be a useful source of dietary anthocyanins. The trait is under the control of the *Pp-1* homoealleles (mapping to each of the group 7 chromosomes) and *Pp3* (on chromosome 2A). Here, *TaMyc1* was identified as a likely candidate for *Pp3*. The gene encodes a MYC-like transcription factor. In genotypes carrying the dominant *Pp3* allele, *TaMyc1* was strongly transcribed in the pericarp and, although at a lower level, also in the coleoptile, culm and leaf. The gene was located to chromosome 2A. Three further copies were identified, one mapping to the same chromosome arm as *TaMyc1* and the other two mapping to the two other group 2 chromosomes; however none of these extra copies were transcribed in the pericarp. Analysis of the effect of the presence of combinations of *Pp3* and *Pp-1* genotype on the transcription behavior of *TaMyc1* showed that the dominant allele *Pp-D1* suppressed the transcription of *TaMyc1*.

## 1. Introduction

The anthocyanins represent a class of secondary metabolites synthesized by most higher plants. They are responsible for the pigmentation of flowers and fruits, and function as attractors for the vectors of pollen and seeds. Their presence in vegetative tissue is associated with the response to biotic and abiotic stress [1,2], enabled by their ability to neutralize free radicals, chelate heavy metal ions, and aid in osmoregulation and photoprotection [1,2,3,4,5,6,7]. In addition, their inclusion in the human diet is beneficial in numerous ways [8,9,10,11,12,13]. The main source of dietary anthocyanins is berries and fruits, but in recent years, cereals are also being considered as additional sources of these compounds [14,15,16,17,18,19].

In bread wheat (*Triticum aestivum* L., *2n* = *6x* = 42, BBAADD) grain the anthocyanins reside either in the pericarp or in aleurone layer; the grain of some accessions has a purple or blue appearance as a result of the anthocyanin content of one or other of these tissues [20]. The genetic basis of purple grain pigmentation resides in the action of the homoeallelic *Pp-1* genes and *Pp3* [21,22,23,24,25]. The former map to the short arms of the homeologous group 7 chromosomes [21,22,23,24,25], and the latter to chromosome arm 2AL [21,22,23]. Comparative mapping has shown that the *Pp-1* genes are orthologs of both maize *C1* and rice *OsC1*, which encode MYB-like transcription factors (TFs) responsible for the activation of structural genes encoding various enzymes participating in anthocyanin synthesis [26,27,28] (Supplementary Table S1). Similarly, *Pp3* has been shown to be orthologous to both *Pb*/*Ra* in rice [29,30] and *Lc*/*R* in maize [31], which encode MYC-like TFs underlying the regulation of anthocyanin synthesis (Supplementary Table S1). Regulatory role of the *Pp* genes has been confirmed by functional analysis of the anthocyanin synthesis structural genes in wheat near-isogenic lines (NILs) differing by the allelic state of the *Pp-1* and *Pp3* genes (both genes were in dominant or recessive state) [32]. Here, the nucleotide sequence of *Pp3* has been determined, and a functional characterization of the gene has been described.

## 2. Results

### 2.1. Identification and Chromosome Location of Wheat Myc-Like Sequences

**Figure 1:**
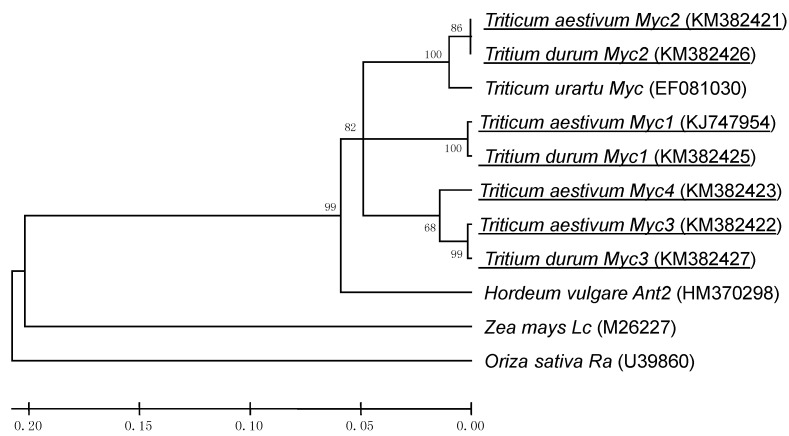
*Myc*-like sequence phylogeny. The sequences shown underlined were isolated in the current study, while the remainders were downloaded from GenBank.

**Figure 2:**
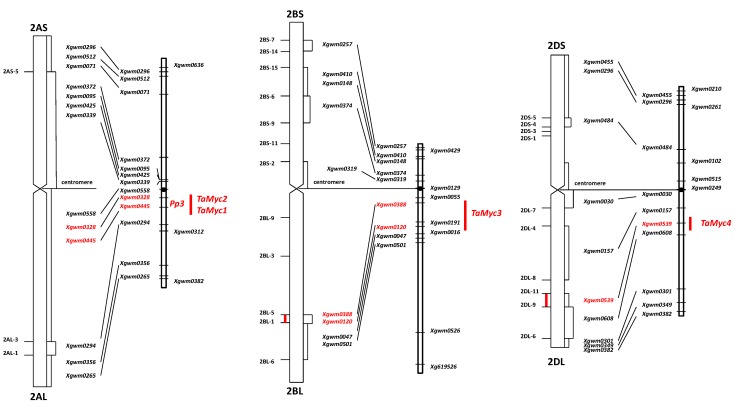
Chromosome location of wheat *Myc* genes.

The BLAST search based on the maize *Lc* and rice *Ra* sequences identified a matching sequence on *T. urartu* BAC clone 404H6 (GenBank accession number EF081030, Supplementary Figure S1), and this sequence allowed the design of a wheat primer pair targeting the *Myc*-like sequences (Supplementary Table S2, Figure S1 and Figure S2). When gDNA from the NIL “i:S29*Pp-A1Pp-D1Pp3*^P^” (Table 1) was amplified using this primer pair, four distinct sequences were generated (Supplementary Figure S3). The pair-wise level of homology between the four sequences varied from 86.7% to 95.8% (Supplementary Table S3 and Figure S3). Three distinct sequences were amplified from *T. durum* gDNA in the same way. The eight sequences (four from *T. aestivum*, three from *T. durum* and one from *T. urartu*) formed three clusters: one grouped *TaMyc1* and *TdMyc1*, the second *TaMyc2*, *TdMyc2* and the *T. urartu* sequence, and the third *TaMyc3*, *TdMyc3* and *TaMyc4* (Figure 1). The sequence information was used to design a series of copy-specific primer pairs (Supplementary Table S2), which when applied to the aneuploid stocks of cv. “Chinese Spring”, allowed *TaMyc1* and *TaMyc2* to be assigned to chromosome arm 2AL, *TaMyc3* to chromosome arm 2BL and *TaMyc4* to chromosome arm 2DL (Figure 2, Supplementary Figure S4).

**Table 1:** Genetic stocks used to characterize the transcription of the *Myc*-like genes in the wheat grain pericarp (controlled by *Pp3* and *Pp-1*), the leaf (*Plb*), the culm (*Pc*) and the coleoptile (*Rc*). D: dominant allele, R: recessive allele, NIL: near-isogenic line, *, **: genotypes, in which the pericarp is, respectively, dark and light purple.

Name	Alternative Name	Description	*Pp*			*Plb*			*Pc*			*Rc*			References
			*-A1*	*-B1*	*-D1*	*-A1*	*-B1*	*-D1*	*-A1*	*-B1*	*-D1*	*-A1*	*-B1*	*-D1*	
► i:S29 *Pp-A1pp-D1pp3*	“Saratovskaya 29” (“S29”)	Russian spring wheat	D	R	R	D	R	R	D	R	R	D	R	R	[25,33]
► i:S29 *Pp-A1Pp-D1Pp3*^PF^*	i:S29 *Pp1Pp2*^PF^	wheat NIL developed on “S29”, donor—“Purple Feed”	D	R	D	D	R	D	D	R	D	D	R	D	[21,24,25]
► i:S29 *Pp-A1Pp-D1Pp3*^P^*	i:S29 *Pp1Pp3*^P^	wheat NIL developed on “S29”, donor—“Purple”	D	R	D	D	R	D	D	R	D	D	R	D	[21,24,25]
► i:S29 *Pp-A1pp-D1Pp3*^PF^**	no	wheat NIL developed on “S29”, donor—“Purple Feed”	D	R	R	D	R	R	D	R	R	D	R	R	[25]
► i:S29 *Pp-A1Pp-D1pp3*^PF^	no	wheat NIL developed on “S29”, donor—“Purple Feed”	D	R	D	D	R	D	D	R	D	D	R	D	[25]
► i:S29 *Pp-A1pp-D1Pp3*^P^**	no	wheat NIL developed on “S29”, donor—“Purple”	D	R	R	D	R	R	D	R	R	D	R	R	[25]
► i:S29 *Pp-A1Pp-D1pp3*^P^	no	wheat NIL developed on “S29”, donor—“Purple”	D	R	D	D	R	D	D	R	D	D	R	D	[25]
◄ i:S29 *pp-A1pp-D1pp3*	line 140;“S29” (“YP” 4D*7A)	wheat NIL developed on “S29”, donor—“Yanetzkis Probat”	R	R	R	R	R	R	R	R	R	R	R	R	[25,33]
“Novosibirskaya 67” (“N67”)	no	Russian spring wheat	R	R	D	R	R	D	R	R	D	R	R	D	[24,34]
“Purple”*	no	Australian spring wheat “k-46990”	R	R	D	R	R	D	R	R	D	R	R	D	[24]
“Purple Feed”*	no	Canadian spring wheat “k-49426”	R	R	D	R	R	D	R	R	D	R	R	D	[24]

► names for NILs obtained on “Saratovskaya 29” with dominant alleles *Pp-D1* and/or *Pp3* inherited from cultivars “Purple” (P) or “Purple Feed” (PF); in these lines dominant allele of *Pp-A1* is from “Saratovskaya 29”; ◄ name for NIL obtained on “Saratovskaya 29” with its own recessive alleles *pp-D1* and *pp3* and recessive *pp-A1* inherited from “Yanetzkis Probat”.

### 2.2. Functional Activity of the Wheat Myc Gene Copies

**Figure 3:**
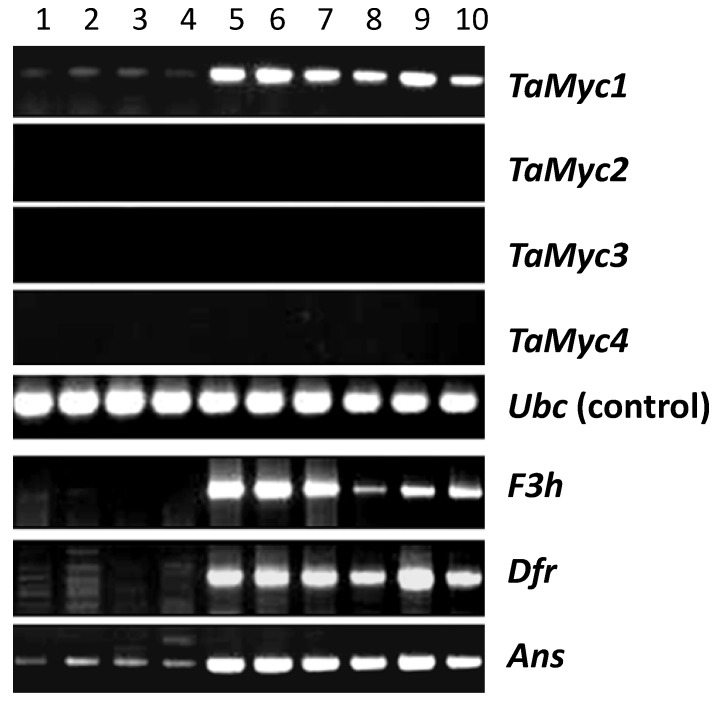
Transcription of the *Myc* gene copies in the pericarp of cv. “Novosibirskaya 67” (**1**); cv. “Saratovskaya 29” (**2**–**4**); “i:S29*Pp-A1Pp-D1Pp3*^P^” NIL (**5**–**6**); “i:S29*Pp-A1Pp-D1Pp3*^PF^” NIL (**7**,**8**); cv. “Purple” (**9**) and cv. “Purple Feed” (**10**).

The transcript abundance of each *Myc* gene in the grain pericarp was derived by RT-PCR. Only *TaMyc1* was strongly represented in the pericarp transcriptome of genotypes harboring dominant alleles at *Pp-1* and *Pp3*. The transcription profile of *TaMyc1* was similar to that of the anthocyanin synthesis pathway genes encoding for flavanone 3-hydroxylase (F3H), dihydroflavonol-4-reductase (DFR) and anthocyanidin synthase (ANS) (Figure 3).

**Figure 4:**
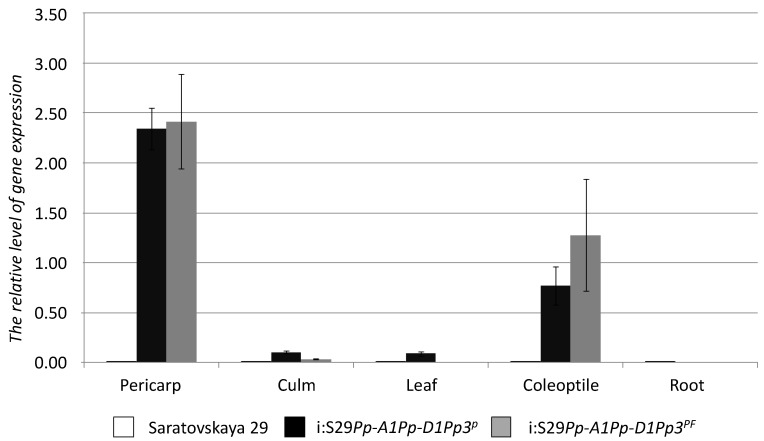
Transcription of *TaMyc1* in various parts of the wheat plant. Statistical analysis of transcript abundances given in Supplementary Table S4.

The quantitative RT-PCR analysis showed that *TaMyc1* transcript was more abundant in both NILs “i:S29*Pp-A1Pp-D1Pp3*^P^”, “i:S29*Pp-A1Pp-D1Pp3*^PF^” than in the parental cultivar “Saratovskaya 29” in all tissues investigated, while the level present in the pericarp was two-three orders of magnitude higher than elsewhere in the plant (Figure 4).

### 2.3. The Full-Length Sequence of TaMyc1

**Figure 5:**
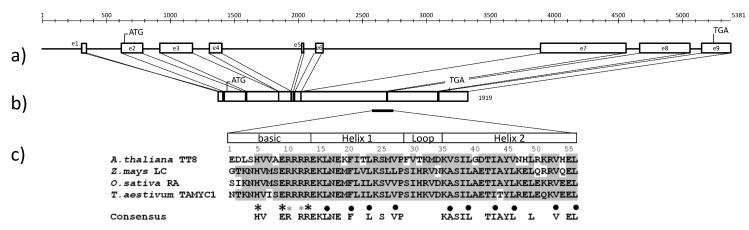
(**a**) Gene structure of *TaMyc1*; (**b**) mRNA identified in the pericarp of the NIL “i:S29*Pp-A1Pp-D1Pp3*^P^”; (**c**) The conserved bHLH domain. The translation start site (ATG) and stop codon (TGA). Black asterisks: amino acid contacts with nucleotide bases, small gray asterisks: amino acid contacts with DNA backbone, dots: non-polar residues important for protein–protein interactions.

Sets of overlapping amplicons were generated to obtain the full sequence of the *TaMyc1* copy present in the NIL “i:S29*Pp-A1Pp-D1Pp3*^P^” (Supplementary Figure S2). The 5381 nt sequence was shown via a comparison of the gDNA and cDNA sequences to be split into nine exons (Figure 5a). The first intron lay in the 5' untranslated region as was determined by 5'RACE method. The length of the open reading frame was 1707 nt, and the predicted product was a 568 residue protein (Figure 5b) harboring a conserved basic helix-loop-helix (bHLH) domain encoded by exons 7 and 8 (Figure 5c).

The bHLH domain consisted of 56 residues, split into a 13 residue segment dominated by basic amino acids and a longer segment predicted to form two amphipathic α helices separated by a 6 residue loop. The basic region contained the conserved residues H^5^-E^9^-R^13^, thought to be critical for DNA binding [35]. The highly conserved hydrophobic residues in helix 1 and 2 are believed to be necessary for achieving the dimerization of a pair of bHLH proteins [35]. An alignment of MYC-like proteins participating in anthocyanin synthesis revealed that they all (including that encoded by the *TaMyc1* gene) shared, in addition to their bHLH domain, a highly conserved run of 200 residues at their N terminal end (Supplementary Figure S5). This segment has been implicated as being important for the proteins’ interaction with R2R3-MYB TFs [36]. The structure of *TaMyc1*, the position of its bHLH domain and the presence of certain other conserved regions are all consistent with its involvement in the regulation of anthocyanin synthesis [35,37].

### 2.4. TaMyc1 Transcription as Affected by the Combination of Pp Alleles Present

**Figure 6:**
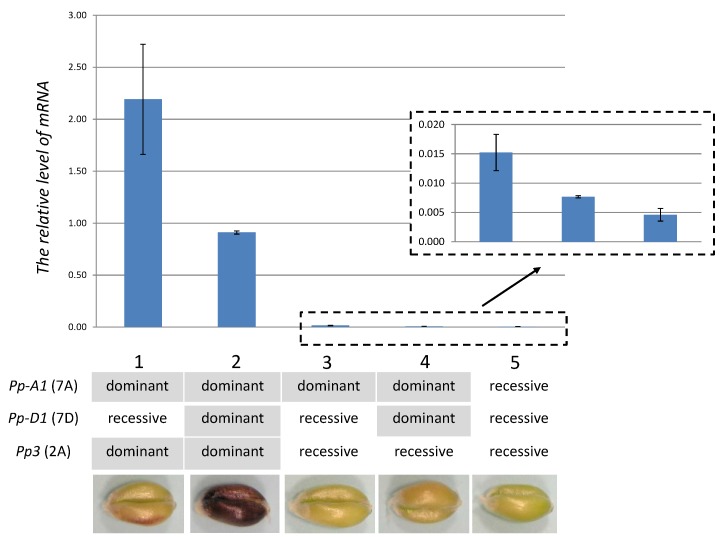
*TaMyc1* transcription in the pericarp of NILs carrying various combinations of *Pp* alleles. 1: “i:S29*Pp-A1pp-D1Pp3*^P^” NIL, 2: “i:S29*Pp-A1Pp-D1Pp3*^P^” NIL, 3: cv. “Saratovskaya 29” (“i:S29*Pp-A1pp-D1pp3*”), 4: “i:S29*Pp-A1Pp-D1pp3*^P^” NIL, 5: “i:S29*pp-A1pp-D1pp3*” NIL. Statistical analysis of transcript abundances given in Supplementary Table S5. The phenotypes of lines set generated on “Purple” as a donor of *Pp* genes are shown.

Genotypes carrying dominant *Pp3* (lines 1, 2, Figure 6) were associated with the most abundant *TaMyc1* transcript, consistent with the notion that *TaMyc1* is synonymous with *Pp3*. In genotypes harboring the dominant allele at *Pp-D1*, the abundance of *TaMyc1* transcript was significantly lower than in those carrying the recessive allele (lines 1, 2, Figure 6). In lines with recessive allele at *Pp3*, dominant *Pp-D1* also reduced the abundance of *TaMyc1* transcript (lines 3, 4, Figure 6). The lowest level of *TaMyc1* transcript was observed in the line bearing recessive alleles at *Pp3* and both *Pp-A1* and *Pp-D1* (line 5, Figure 6). Described pattern of expression of the *TaMyc1* gene was also observed for the complete lines set generated on cv. “Purple Feed” as a donor of the *Pp* genes (Supplementary Table S6). These data suggested that the presence of the dominant allele at *Pp-D1* had an incomplete suppressive effect on the level of *TaMyc1* transcription.

## 3. Discussion

The chromosomal location (Figure 2) and transcription profile (Figure 3 and Figure 4) of *TaMyc1* were all consistent with the notion that it is *Pp3*, one of the two complementary *Pp* genes required for the synthesis of anthocyanins in the pericarp. This gene has high level of structural similarity with the others genes (Figure 5, Supplementary Figure S5), that have been shown to encode bHLH transcriptional factors, participating in anthocyanin synthesis regulation, such as maize *Lc* and *B*, rice *Ra* and *Pb*, *Arabidopsis TT8*, barley *Ant2* [29,30,31,38,39,40]. Partial sequences of the other three *Myc* copies identified all shared a high level of sequence similarity with *TaMyc1* (Figure 1, Supplementary Figure S3 and Table S3), but none of them was transcribed in the pericarp (Figure 3). Plant genomes typically harbor large numbers of bHLH-domain containing TFs, thought to have evolved via multiple duplication events followed by functional specialization [29,41,42].

The *TaMyc1* sequence is more similar to that of *T. durum Myc1* than it was to that of the A genome donor gene *TuMyc*. Assuming that *TaMyc1* and *TdMyc1* are orthologous, the implication is that *TaMyc2* and *TdMyc2* are their respective paralogs; the latter are directly related by descent to *TuMyc*. The purple grain trait has not been noted to date in any A genome diploid species [43]. As a result, it is likely that *TdMyc1* arose later than *TdMyc2* via a segmental duplication event post the formation of the BA tetraploid, and that *TaMyc1* and *TaMyc2* were transmitted from the BA tetraploid to the BAD hexaploid.

Although the functions of *TaMyc2* through *TaMyc4* have not been identified, it is possible that one or more of them do participate in anthocyanin synthesis, perhaps outside the pericarp, or in response to an external stimulus. In a number of plant species, anthocyanin synthesis is regulated by TF complexes [44,45]. The transgenic activation of the maize anthocyanin synthesis structural gene *Bz1* requires the presence of both *C1* (an R2R3-MYB TF) and *B* (a bHLH TF) [36]. Similarly, in *Petunia hybrida*, the TFs AN2 and PhJAF13 co-regulate a number of anthocyanin synthesis genes [37]. In wheat, anthocyanin synthesis in the culm, leaf and coleoptile is under the control of genes thought to be MYB family TFs [27]. *TaMyc1* was up-regulated in the coleoptile (Figure 4), which implies that it may interact with the *Myb* gene *Rc*.

Anthocyanin biosynthesis in grain pericarp is controlled by two complementary *Pp* genes, which encode for R2R3-MYB and bHLH TFs. The pericarps of genotypes harboring the dominant allele at both *Pp-1* (*R2R3-Myb*) and *Pp3* (*Myc/bHLH*) genes are pigmented from light to dark purple [25]. The presence of *Pp-A1*, inherited from cv. “Saratovskaya 29”, in combination with *Pp3*, inherited from cvs “Purple” or “Purple Feed”, ensures that the pericarp of each of the NILs “i:S29*Pp-A1pp-D1Pp3*^P^” and “i:S29*Pp-A1pp-D1Pp3*^PF^” is light purple in color, whereas the color of both “i:S29*Pp-A1Pp-D1Pp3*^P^” and “i:S29*Pp-A1Pp-D1Pp3*^PF^” is dark purple due to the presence of *Pp-D1* [25].

The transcription behavior of *TaMyc1* varied according to the plant's *Pp* gene content (Figure 6). The highest abundance was noted in the combination *Pp3* + *Pp-A1*, although this feature was unrelated to the intensity of pericarp pigmentation, since the relevant NIL produced a light purple rather than a dark purple pericarp. The most intense pigmentation was seen in the combination *Pp3* + *Pp-A1* + *Pp-D1*, although in this case, the abundance of *TaMyc1* transcript was only about half of that observed in the *Pp3* + *Pp-A1* combination (Figure 6, Supplementary Table S5 and Supplementary Table S6). A similar interaction was noted where the recessive allele of *Pp3* was present; in this case the overall levels of *TaMyc1* transcript were much reduced (Figure 6, Supplementary Table S5 and Supplementary Table S6). The conclusion is that there is an influence exerted by the *Pp-D1* genes on *TaMyc1* expression, but the nature of the underlying mechanism is obscure. A possible model might involve negative feedback, in which the presence of an active R2R3-MYB/bHLH/WD40 (MBW) complex represses the transcription of *TaMyc1* and leads to optimal proportion of partners in functional MBW complex.

Negative and positive feedback regulation of anthocyanin synthesis has also been reported in *Arabidopsis thaliana* [45]. Expression of the *TT8* gene has been shown to be positively regulated by MBW complex including the WD40 TTG1, the MYBs TT2/PAP1 and the bHLHs TT8 itself or GL3/EGL3 [46]. In addition to this positive feedback regulation two negative regulators of anthocyanin synthesis were identified (MYBL2 and CPC), both of which encode single MYB repeat proteins [47,48]. Although both MYBL2 and CPC inhibit anthocyanin accumulation by repressing the biosynthesis genes [47,49], direct suppression of the *Myb* and *bHLH* regulatory genes expression has been also reported for MYBL2 [47].

## 4. Experimental Section

### 4.1. Plant Materials

*Myc*-like sequences were identified and isolated from the near-isogenic line (NIL) “i:S29*Pp-A1Pp-D1Pp3*^P^” (Table 1) and from *T. durum* accession TRI15744; the latter was obtained from the IPK genebank in Gatersleben (Germany). The chromosomal and intra-chromosomal locations of the wheat sequences obtained were assigned using nulli-tetrasomic, ditelosomic, and deletion lines of cv. “Chinese Spring” [50,51,52]. The other genetic stocks used to profile the transcription of the *Myc* genes are listed in Table 1.

### 4.2. Gene Identification, Isolation and Sequence Analysis

The maize *Lc* (GenBank accession M26227) and rice *Ra* (U39860) sequences were used as a query to identify a *Myc*-like sequence present on a *T. urartu* bacterial artificial chromosome (BAC) clone. A pair of PCR primers (pair 1: sequences given in Supplementary Table S2) was designed to amplify a segment of this gene, and was then used to recover its *T. aestivum* homologs via a PCR based on DNA extracted from fresh leaves following [53]. These and all subsequent primers were designed using OLIGO software [54]. Amplification of gDNA templates from the NIL “i:S29*Pp-A1Pp-D1Pp3*^P^” was performed in 20 µL PCRs each containing 1 U *Taq* DNA polymerase (Medigen, Novosibirsk, Russia), 1× PCR buffer (Medigen), 1.5 or 1.8 mM MgCl_2_ (Supplementary Table S2), 0.2 mM dNTP and 0.25 µM of each primer. Amplification by different primer pairs was performed in distinct PCR conditions and amplification regimes (Supplementary Table S2). The amplified fragments were purified from a 1% agarose gel, using a DNA Clean kit (Cytokine, St. Petersburg, Russia), then cloned using a PCR Cloning kit (Qiagen, Venlo, The Netherlands). Ten clones were sequenced in both directions to exclude any PCR and/or sequencing errors. The full length *TaMyc1* sequence present in the NIL “i:S29*Pp-A1Pp-D1Pp3*^P^” was re-constructed from a series of overlapping amplicons covering the relevant stretch of genomic DNA, using primer sequences designed from the sequences of contigs 249890, 467773, 1475001 and 1821237 (http://www.cerealsdb.uk.net) [55].

A Mint RACE primer set (Evrogen, Moscow, Russia) was used to obtain the ends of *TaMyc1* transcripts present in the grain pericarp. Two rounds of 5' and 3' end amplification were conducted (primers listed in Supplementary Table S2). The resulting amplicons were cloned using a Qiagen PCR Cloning kit; a total of respectively, 35 and 12 clones obtained from the 5'- and 3'-RACE were sequenced in both directions. DNA sequencing was performed by SB RAS Genomics (Novosibirsk, Russia, http://sequest.niboch.nsc.ru). Multiple sequence alignments were carried out using Multalin v5.4.1 software [56], and the subsequent phylogenetic analysis using MEGA v5.1 software [57], based on the Neighbor-Joining algorithm and 1000 bootstrap replicates. Gene structure was determined using the FGENESH+ program [58] and confirmed by sequencing cDNAs obtained from the grain pericarp (Supplementary Table S2).

### 4.3. Chromosomal Assignment of Wheat Myc Sequences

Amplification of gDNA templates from cv. “Chinese Spring” and its aneuploid derivates was performed in 20 µL PCRs each containing 1 U *Taq* DNA polymerase (Medigen, Novosibirsk, Russia), 1× PCR buffer (Medigen), 1.5 or 1.8 mM MgCl_2_ (Supplementary Table S2), 0.2 mM dNTP and 0.25 µM of each primer. The amplification was initiated by a denaturing step (94 °C/2 min), followed by 13 cycles of 94 °C/15 s, 65 °C/30 s (decreasing by 0.7 °C/cycle), 72 °C/45 s, 24 cycles of 94 °C/15 s, 56 °C/30 s, 72 °C/45 s and completed with a final extension step of 72 °C/5 min.

### 4.4. Transcription Analysis

A ZR Plant RNA MiniPrep^TM^ kit (Zymo Research, Irvine, CA, USA) followed by DNAse treatment was employed to extract RNA from the grain pericarp, leaf, culm, coleoptile, and root of genotypes described in Table 1. Plants and seedlings for RNA extractions were grown, respectively, in greenhouse (ICG Greenhouse Core Facilities, Novosibirsk, Russia) or in climatic chamber “Rubarth Apparate” (RUMED GmbH, Laatzen, Germany) under 12 h of light per day at 20–25 °C. Pericarp samples for RNA extraction were peeled by scalpel from immature grains within 55th–75th day after sowing. RNA from leaf and culm were extracted within 70th–75th day after sowing. RNA samples from roots and coleoptiles were obtained on the fifth day after caryopsis germination. Single-stranded cDNA was synthesized in a 20 µL reaction from a template consisting of 0.7 μg total RNA using a (dT)15 primer and a Fermentas RevertAid™ first strand cDNA synthesis kit (Fisher Scientific, Loughborough, UK). Subsequent RT-PCRs were primed either with *Myc* copy-specific primers (Supplementary Table S2) or with the primers amplifying a segment of the genes *F3h*, *Dfr* and *Ans* [32]. A fragment of the wheat *Ubc* sequence (X56601) was used as the internal reference [59]. The PCR conditions and amplification regime were as above (Section 4.3), and the amplicons obtained were electrophoresed through 2% agarose gels. Quantitative RT-PCRs (qPCRs) were based on a SYBR Green I kit (Syntol, Moscow, Russia). Pre-determined quantities of cloned cDNA were used to generate a standard curve. Three biological replicates for each sample were run as three technical replicates. Differences in transcript abundance between lines were tested by applying the Mann-Whitney *U*-test [60], adopting a significance threshold of *p* ≤ 0.05.

## Supplementary Materials

Supplementary materials can be accessed at:http://www.mdpi.com/1420-3049/19/12/20266/s1.
