# Supplementary material for: The Regulation of Anthocyanin Synthesis in the Wheat Pericarp
Source: Molecules. 2014 Dec 4;19(12):20266–79. doi: 10.3390/molecules191220266 (PMC6271175; doi:10.3390/molecules191220266)
Supplement: Supplementary file 1 [file molecules-19-20266-s001.pdf]

Supplementary Materials

Table S1. Genes encoding for transcription factors participating in anthocyanin biosynthesis regulation in different plant species.

	MYC (bHLH)	MYB	WD40
Arabidopsis thaliana	*TT8* (Nesi *et al.*, 2000) *GL3/EGL3* (Bernhardt *et al.*, 2003; Heim *et al.*, 2003; Zhang *et al.*, 2003)	*TT2*, *CPC* (Borevitz *et al.*, 2000; Gonzalez *et al.*, 2008) *MYBL2* (Dubos *et al.*, 2008; Matsui *et al.*, 2008) *PAP1*, *PAP2* (Borevitz *et al.*, 2000)	*TTG1* (Walker *et al.*, 1999)
Petunia hybrida	*AN1*, *JAF13* (Llyod *et al.*, 1992; Quattrocchio *et al.*, 1998; Spelt *et al.*, 2000, 2002)	*AN2*, *AN4* (Quattrocchio *et al.*, 1999, 2006)	*AN11* (de Vetten *et al.*, 1997)
Antirrhinum majus	*Delila* (Gong *et al.*, 1999)	*Rosea1*, *Rosea2*, *Venosa*, *MYB308*, *MYB330* (Schwinn *et al.*, 2006)	not defined
Vitis vinifera	*MYC1*, *MYCA1* (Hichri *et al.*, 2010; Matus *et al.*, 2010)	*MYBA1, MYBA2*, *MYB5a* (Kobayashi *et al.*, 2002; Deluc *et al.*, 2006, 2008; Walker *et al.,* 2007; Cutanda-Perez *et al.*, 2009)	*WDR1*, *WDR2* (Matus *et al.*, 2010)
Zea mays	*B*, *R*, *Lc*, *Sn*, *In1* (Burr *et al.*, 1996; Chandler *et al.*, 1989; Ludwig *et al.*, 1989; Consonni *et al.*, 1993; Petroni *et al.*, 2000)	*C1*, *Pl1*, *P1* (Paz-Ares *et al.*, 1987; Chandler *et al.*, 1989; Goff *et al.*, 1990; Petroni *et al.*, 2000)	*PAC1* (Selinger and Chandler, 1999)
Oryza sativa	*R*, *Pl*, *Ra*, *Pb* (Hu *et al.*, 1996; 2000; Sakamoto *et al.*, 2001; Wang and Shu, 2007)	*OsC1*(Reddy *et al.*, 1998; Saitoh *et al.*, 2004)	not defined
Hordeum vulgare	*ANT2* (Cockram *et al.*, 2010)	not defined	not defined
Triticum aestivum	*TaMyc1* (current study)	*Mpc1* (Li *et al.*, 1999)	not defined

Table S2. PCR primers amplifying *Myc*-like gene sequences of wheat. e: exon, i: intron, UTR: untranslated region, TDN: touchdown, TDN ext: touchdown extended, *: primers designed from *T. aestivum* contigs 32064, 42144, 65805, 248535, 249890, 302985, 445995, 467773, 1280203, 1475001, 1815797, 1821237 and 4203206 found in www.cerealsdb.uk.net. Primer pairs used for cDNA sequencing are shown in gray color.

Primer Pair Number	Purpose	Gene (Copy)	PCR Product Length (DNA/cDNA)	Forward Primer (FP), 5'→3'	Reverse Primer (RP), 5'→3'	PCR Conditions (Amplification Program/$MgCl_2$ Concentration, mM)	Gene Region Containing Primer Annealing Site	
							Forward	Reverse
1	cloning	*TaMyc1-4*	373, 379, 382	gtggacaaggcatccatcctagc	tcttgagagcgaggaggccg	60 grad[1]/1.5	e8	e8
2	sequencing	*TaMyc1*	453/-	ataattgcatcggcaggc *	gaactaagaagtaagctagccatgac *	TDNext[2]/1.8	5' region	i1
3	sequencing	*TaMyc1*	640/228	gctccatcttcctccccg	cgccgttgtagaacccgt	TDN[2]/1.0	e1	e3
4	sequencing	*TaMyc1*	491/-	gtcatggctagcttacttcttagttc *	cacaacaaacggccggt *	55 grad[1]/1.8	i1	i2
5	sequencing	*TaMyc1*	296/-	taccggccgtttgttgt *	tgttcttgagatacatacccttg *	TDNext[2]/1.8	i2	e3i3
6	sequencing	*TaMyc1*	393/262	tcaccagctcggcggaca *	gcgcactgagcattgcacaa *	TDN[2]/1.8	e3	e4
7	sequencing	*TaMyc1*	600/-	gagcaacgagcatgtttggt *	atcgggtgcagattagagagtg *	TDNext[2]/1.8	e4	i4
8	sequencing	*TaMyc1*	147/-	acgtgtagccgcatccttgg *	ccggcagaggtagcatgcat *	TDN[2]/1.8	i4	i4
9	sequencing	*TaMyc1*	245/-	atggcgcccatgcatgc *	aaggagaacatcgtgtgcttcatc *	TDN[2]/1.8	i4	i6
10	sequencing	*TaMyc1*	802/-	tgagttttcgtcccatgatga *	catgagatttgcccctgtaag *	TDNext[2]/1.8	i6	i6
11	sequencing	*TaMyc1*	687/-	cttacaggggcaaatctcatg *	gagaactggaaaaactcatggac *	TDNext[2]/1.8	i6	i6
12	sequencing	*TaMyc1*	492/-	aaatcagtccatgagtttttcca *	cgtgattcgctcaaggttgt *	TDN[2]/1.8	i6	e7
13	sequencing	*TaMyc1*	534/-	gatacccggggaacttgagctag *	ttactttttggtggtgtgcgtgt *	TDNext[2]/1.8	e7	i7
14	sequencing	*TaMyc1*	283/177	aaccatgtcatttcggagagga *	ggccgccctgttggatc	TDN[2]/1.8	e7	e8

Table S2. *Cont.*

Primer Pair Number	Purpose	Gene (Copy)	PCR Product Length (DNA/cDNA)	Forward Primer (FP), 5'→3'	Reverse Primer (RP), 5'→3'	PCR Conditions (Amplification Program/$MgCl_2$ Concentration, mM)	Gene Region Containing Primer Annealing Site	
							Forward	Forward
15	sequencing	*TaMyc1*	422/331	acgacggcctgagcagtg	atgaagggttgccgatagcc *	TDN[2]/1.8	e8	3UTR
16	5'RACE	*TaMyc1*	466/332	agtagttcgtccatgccag	ggtcctcaggcgacag	MINTa[3], 55°C, 29 cycles	e2	e3
17		*TaMyc1*	-/151	(primer for control)	ccacgtcagaacccc	MINTa[3], 53°C, 26 cycles		e2e3
18	3'RACE	*TaMyc1*	531/440	1FP	agtgcttcgctaatcatc	MINTa[3], 60°C, 24 cycles	e8	e9
19		*TaMyc1*	407/316	gcgggaagaaggtgttag	(primer for control)	MINTa[3], 55°C ,20 cycles	e8	
20	mapping expression	*TaMyc1*	283/177	13F	13R	TDN[2]/1.8	e7	e8
21	mapping expression	*TaMyc2*	330/220	13F	tcttcccgccgacttcatga	TDN[2]/1.5	e7	e8
22	mapping expression	*TaMyc3*	347/198	13F	gagttttcggacggctgttg	TDN[2]/1.8	e7	e8
23	mapping expression	*TaMyc4*	341/188	13F	acggctgttccggcca	TDN[2]/1.8	e7	e8

[1] The amplification was initiated by a denaturing step (94 °C/2 min), followed by 45 cycles (35 cycles in **55 grad** program) of 94 °C/15 s, 60 °C (55 °C in **55 grad** program)/30 s, 72 °C/60 s, and completed with a final extension step of 72 °C/5 min; [2] The amplification was initiated by a denaturing step (94 °C/2 min), followed by 13 cycles of 94 °C/15 s, 65 °C/30 s (decreasing by 0.7 °C/cycle), 72 °C/45 s (2 min in **TDNext** program), 24 cycles of 94 °C/15 s, 56 °C/30 s, 72 °C/45 s (2 min in **TDNext** program); and a final extension of 72 °C/5 min; [3] The amplification program was as in a manual to a Mint RACE primer set (Evrogen, Moscow, Russia) with annealing temperature and numbers of cycles, established empirically (pointed in column "PCR Conditions").

Table S3. Sequence similarity between *TaMyc1* and its three related copies.

	TaMyc1	***TaMyc2***	***TaMyc3***
TaMyc2	87.61%<br>2 in/dels (6+3bp)	-	-
TaMyc3	89.97%<br>1 in/del (3bp)	86.73%<br>3 in/del (3+6+3 bp)	-
TaMyc4	91.45%<br>1 in/del (3bp)	87.32%<br>3 in/del (3+6+3 bp)	95.83%<br>0 in/del

Table S4. Fold differences in transcript *TaMyc1* abundance between test genotypes in various parts of the wheat plant. *: differences statistically significant at $p \leq 0.05$.

Comparable Genotypes	Pericarp	Culm	Leaf	Coleoptile	Root
"i:S29*Pp-A1Pp-D1Pp3*^{P}"/"Saratovskaya 29"	1446.31 *	18.79 *	35.54 *	61.12 *	47.87 *
"i:S29*Pp-A1Pp-D1Pp3*^{PF}"/"Saratovskaya 29"	1492.27 *	6.81 *	2.59 *	100.82 *	30.79 *
"i:S29*Pp-A1Pp-D1Pp3*^{P}"/"i:S29*Pp-A1Pp-D1Pp3*^{PF}"	0.97	2.76 *	13.70 *	0.61	1.55 *

Table S5. Fold differences in transcript *TaMyc1* abundance between genotypes marked in the table line to the genotypes marked in the table row. *: differences significant at $p \leq 0.05$.

	**Saratovskaya29<br>i:S29*Pp-A1pp-D1pp3***	**i:S29*Pp-A1Pp-D1Pp3*^{P}**	**i:S29*Pp-A1pp-D1Pp3*^{P}**	**i:S29*Pp-A1Pp-D1pp3*^{P}**
**i:S29*pp-A1pp-D1pp3***	3.31 *	197.82 *	476.45 *	1.67 *
**Saratovskaya29<br>i:S29*Pp-A1pp-D1pp3***	-	59.76 *	143.93 *	-
**i:S29*Pp-A1Pp-D1pp3*^{P}**	1.98 *	118.63 *	285.71 *	-
**i:S29*Pp-A1Pp-D1Pp3*^{P}**	-	-	2.41 *	-

Table S6. Fold differences in transcript *TaMyc1* abundance between genotypes marked in the table line to the genotypes marked in the table row. * differences significant at $p \leq 0.05$.

	**Saratovskaya29<br>i:S29*Pp-A1pp-D1pp3***	**i:S29*Pp-A1Pp-D1Pp3*^{PF}**	**i:S29*Pp-A1pp-D1Pp3*^{PF}**	**i:S29*Pp-A1Pp-D1pp3*^{PF}**
**i:S29*pp-A1pp-D1pp3***	3.31 *	80.55 *	150.01 *	3.04 *
**Saratovskaya29<br>i:S29*Pp-A1pp-D1pp3***	-	24.33 *	45.32 *	-
**i:S29*Pp-A1Pp-D1pp3*^{PF}**	1.09	26.46 *	49.28 *	-
**i:S29*Pp-A1Pp-D1Pp3*^{PF}**	-	-	1.86	-

Figure S1. Multiple alignment of the coding regions of maize *Lc*, rice *Ra* and the *T. urartu Myc*-like gene. Intron positions are marked by triangles, its localizations were predicted by comparison of the mRNA sequences with the corresponding rice (AL606682) and maize (DQ414252) genomic sequences with determined gene structures. The sites of the primers forming primer pair 1 (Table S2) are underlined.

```
Z.mays Lc (M26227)    ATGGCGCTTTCAGCTTCCCGAGTTCAGCAGGCGGAAGAACTGCTGCAACGACCTGCTGAGAGGCAGCTGATGAGGAGCCAGCTTGCTGCAGCCGCCAGGAGCATCAACTGGAGCTACGCCCTCTT
O.sativa Ra (U39860)                                              ATGGAAGA   GACCCCTCTGCCATCCGGGAAGAA CT  TCAGGAGCCAGCTTGCTGCTGCAGCGAGGAGCATCAATTGGACGTATGCCATATT
T.urartu (EF081030)   ATGGCGCTGCCAGTAATTCG   TCCGAGCCAGGAAGAACCCACTCTACCGCCGCCGGCCCGGAAGCAATTCAGCAACCAGCTCGCTGCTGCTGTGAGGAGCATCAACTGGAGCTACGCCATATT
                      5165                                                                                                                    5044

                                              i1
Z.mays Lc (M26227)    CTGGTCCATTTCAGACACTCAACCAG|GGTGCTGACGTGGACGGACGGGTTCTACAACGGCGAGGTGAAGACGCGGAAGATCTCCAACTCCGTGGAGCTGACATCCGACCAGCTCGTCATGCAGA
O.sativa Ra (U39860)  TTGGTCCATTTCAACCAGCCGCCCAG|GAGTTCTGACTTGGAAGGACGGCTTCTACAACGGCGAGATAAAGACGAGGAAGATCACGAACTCCATGAACCTCACGGCCGACGAGCTGGTCCTGCAGA
T.urartu (EF081030)   CTGGTCCGTTTCAACCAGCCGTCCAG|GGTTCTGACGTGGAAGGACGGGTTCTACAACGGCGAGATAAAGACGAGGAAGGTCACCAGCTCGGCGGACCTCACCGCCGACCAGCTCGTCCTACAGA
                      5043                    5017...4890                                                                                     4793

Z.mays Lc (M26227)    GGAGCGACCAGCTCCGGGAGCTCTACGAGGCCCTCCTGTCGGGCGAGGGCGACCGCCGCGCTGCGCCTGCGCGGCCGGCCGGCTCTCTGTCGCCGGAGGACCTCGGCGACACCGAGTGGTACTAC
O.sativa Ra (U39860)  GAAGCGAGCAGCTGAGGGAGCTCTACGACTCTCTCCTCTCCGGCGAGTGCGGCCACCGAGC GAG     GAGGCCCGTCGCTGCGCTGTTGCCGGAAGATCTCGGGGACACGGAATGGTACTAC
T.urartu (EF081030)   GGAGCGAGCAGCTGCGGGAGCTCTACGAGTCTCTCCTCTCCGGCCAGTGCGACCACCGGGCCAGG      CGTCCAGCCGCCGCGCTGTCGCCGGAGGACCTCGGGGACGCCGAGTGGTACTAC
                      4792                                                                                                                    4674

                                                      i2
Z.mays Lc (M26227)    GTGGTCTCCATGACCTACGCCTTCCGGCCAGGCCAAG|GTTGCCCGGCAGGAGTTTCGCGAGCGACGAGCATGTCTGGCTGTGCAACGCGCACCTCGCCGGCAGCAAAGCCTTCCCCCGCGCGCT
O.sativa Ra (U39860)  GTCGTCTGCATGACCTACGCCTTCGGCCCCGGCCAAG|GTTGCCAGGCAAAAGCTTCGCAAGCAATGAATTTGTTTGGCTGACAAACGCTCAGTCTGCAGATAGAAAACTATTCCATCGCGCGCT
T.urartu (EF081030)   ACGGTCTGCATGAGCTACGCCTTCCGCCCTGGCCAAG|GTTGCCAGGCAGAAGCTTTGCGGGCAACGAGCATGTTTGGCTGTGCAATGCTCAGTGCGCAGACACGAAAACCTTCCAGCGCGCACT
                      4673                               4636...3560                                                                          3474

                              i3           i4                                                      i5
Z.mays Lc (M26227)    CCTGGCCAA|GAGCGCGTCCATTCA|GTCAATCCTCTGCATCCCGGTTAT   GGGCGGCGTGCTTGAGCTTGGTACAACTGACAC|GGTGCCGGAGGCCCCGGACTTGGTCAGCCGAGCAACCGCGG
O.sativa Ra (U39860)  TATAGCAAA|GAGTGCATCTATTAA|GACAATCGTCTGCGTGCCATTTATCATGCATGGTGTCCTTGAGCTCGGGACCACTGATCC|GATTTCGGAGGACCCGGCTCTCGTCGACCGTATCGCGGCGT
T.urartu (EF081030)   CTTAGCAAA|GACGGCGTCTATCCA|GACAGTCGCCTGCATTCCCTTAAT   GGGTGGTGTGCTTGAGCTCGGGACAACAGATAC|CGTTTTGGAAGACATGGGCATGGTGAACCGGATCAGAACAT
                      3473      3464...3329 3315...3221                                          3165...1201                                  1162

Z.mays Lc (M26227)    CTTTCTGGGAGCCGCAGTGCCCGAGCTCCAGCCCGTCAGGACGAGCAAACGAGACCGGCGAGGCCGCAGCAGACGACGGCACGTTTGCGTTCGAGGAACTCGACCACAATAATGGCATGGACGAC
O.sativa Ra (U39860)  CGTTCTGGGATACGCCGC CCCGCGC    GGCGTTCTCGTCG        GAGGCGGGAGACGCCGACATCGTCGTC              TTCGAAGACCTCGACCATGGCAACGCCGCCGTCGAA
T.urartu (EF081030)   CTTTCTGGGACTTGAAGTTCCCAA        CATGCTCGAAGT CGGAGGAGCCGAACTCCAGCCCATCAGTAGACGA              TACTGGGGACGCTGACAT        CGTGTTCGAT
                      1161                                                                                                                    1064

Z.mays Lc (M26227)    ATAGAGGCGATGACCGCCGCCGGGG GACACGGGCAGGAGGAGGAG CTAAGACTAAGAGAAGCCGAGGCCCTGTCAGACGACGCAAGCCTGGAGCACATCACCAAGGAG   ATCGAGGAGTTC
O.sativa Ra (U39860)  GCGACGACGACGACGGTCCCGGGGAGCCACACGCGGTAGCCGGCGGCGAGGTCGCCGAGTG  CGAGCCCAACGCCGACAACGA   CCTCGAGCAGATCACCATGGACGACATCGGCGAGCTC
T.urartu (EF081030)   GT  CCTCAAACATGACACCATGG   CCACGATGATAACCAGGGAACTTGAGCCAGGGGAGGTTGAGTGCCTATCCGATGGCAA   CCTCGAGCGGATCACGAAGGAG   ATCAACGGGTTC
                      1063                                                                                                                    950

Z.mays Lc (M26227)    TACAGCCTCTGCGACGAAATGGACCTGCAGGCGCTACCACTACCGCTAGAGGA CGGCTGGAC   CGTGGAC       GCGTCCAATTTCGAGGTCCCCTGCTCTTCCCCGCAGCCAGCGCCGC
O.sativa Ra (U39860)  TACAGCCTCTGCGAAGAGCTGGACGT CGTGCGCCCTCTCGACGACGACAGTAGCAGCTGGGCGGTCGCGGATCCCTGGTCGTCCTTTCAGCTAGTTCTGACGTCTTCTCCGGCGCCGGATCAGG
T.urartu (EF081030)   TACGGCCTCTGCGATGAGCTGGATGTGGGGGC    TCTCGAGGAAAACTGGATCA  TGGGCGG                GTC   TTTCGAGGTCATG   TCTTC       GCCGGAAGCGC
                      949                                                                                                                     859
```

Figure S1. *Cont.*

```
Z.mays Lc (M26227)      CTCCGGTGGACAGGGCTAC   CGCTAACGTCGCCGCCGACGCCTC     AAGGGCAC CCGTCTACGGCTCTCGCGCGACGAGTTTCATGGCTTGGACGAGGTCCTCGCAGCAGTCGTCGTGC
O.sativa Ra (U39860)    CGCCGGCGGCGGAAGCTACTGACGTCGACGACGTCGTCGTCGCCGCTTTAGACGGTAGCTCCATTGATGGATCTTGCAGGCCG   TCGCCGTCGAGTTTTGTGGCGTGGAAGAGGACGGCGGAC
T.urartu (EF081030)     CGCCAGCGCCTGTCGCCACGGACGGGGTCACTAGTGGTGTTGTTGCTTT      TAGTTCCGTTGGACCCTCTCAC            CCATCGTGCTTTGCGGCTTGGAAGAGATCATGGCAC
                        858                                                                                                                         752

Z.mays Lc (M26227)      TCCGACGACGCGGCGCCCGCAGCAGTAGTGCCGGCCATCGAGGAGCCGCAGAGATTGCTGAAGAAAGTGGTGGCCGGCGGCGGTGCTTGGGAGAGCTGTGGCGGC GCGACGGGAGCAGCACAGG
O.sativa Ra (U39860)    TCGGACGAGGTGCAGGCCG TGCCGCTCATCAG   CGGAGAGCCGCCACAGAAGTTGCTGAAGAAAGCTGTCGCCGGAGCCGGTGCCTGGATGAACAATGGTGACAGCAGCGCG GCGGCGATGA
T.urartu (EF081030)     TCGGCCAAAGACGTG         GTTGTGCCGGTCGCCGGGGAGTCGCAGAAATTGCTAAAGAAAGCTTTGGCCGGTG   GGGCATGGGCAAATAATGATGACGACGGCA    CCGCTAGAG
                        751                                                                                                                         643

                                                                                                                                     i6
Z.mays Lc (M26227)      AAATGAGTGGCACTGGCACCAAGAACCACGTCATGTCGGAGCGAAAGCGACGAGAGAAGCTCAACGAGATGTTCCTCGTCCTCAAGTCACTGCTTCCGTCCATTCACAGGGTGAACAAAGCGTCG
O.sativa Ra (U39860)    CGACTCAAGAAAGCAGCATCAAGAACCATGTCATGTCAGAGAGAAGGCGCCGGGAGAAGCTCAACGAGATGTTCCTGATTCTCAAATCAGTTGTCCCGTCCATTCACAAGGTGGACAAAGCATCC
T.urartu (EF081030)     CTCAGGAAAGTACCAACACCAAAAACCATGTCATTTCGGAGAGGAGGCGCCGGGAGAAGCTCAACGAGATGTTTCTGATTCTTAAGTCGTTGGTCCCGTCCATTCACAAGGTGGACAAGGCATCC
                        642                                                                                                         533...420       406
                                                                                                                                               1FP

Z.mays Lc (M26227)      ATCCTCGCCGAAACGATAGCCTACCTCAAGGAGCTTCAGAGAAGGGTGCAAGAGCTGGAGTCCAGTAGGGAACCTGCGTCGCGCCCATCCGAAACGACGACAAGGCTAATAA CAAGGCCCTCCC
O.sativa Ra (U39860)    ATTCTCGCAGAAACGATAGCCTACCTCAAAGAGCTGGAGAAAAGAGTGGAAGAGCTGGAATCCAGCAGCCAACCATCGCCATGTCCATTGGAAACAAGAAGCAGGCGAAAGTGCCGTGAGATCAC
T.urartu (EF081030)     ATCCTAGCTGAGACGATAGCATATCTCAGAGAGCTGGAACAGAAGGTGGAGGAGCTAGAATCTAACAGGG               CTGCCGGAACAAAAGTTGTCAGGAAACGTCATGAAGTTGG
                        405                                                                                                                         296
                         1FP

Z.mays Lc (M26227)      GTGGCAATAA TGAGAGTGTGAGGAAGGAGGTCTGCGCGGGCTCCAAGAGGAAGAGCCCAGAGCTCGGCAGAGACGACGTGGAGCGCCCCCCGGTCCTCACCATGGACGCCGGCACCAGCAACGT
O.sativa Ra (U39860)    TGGGAAGAAGGTTTCTGCAGGAGCGAAGAGAAAGGCGCCGGCGCC    GGAGGTGGCCAGCGA CGACGACACCGACGGGGAGCG           GCGCCATTGT GTGAGCAACGTGAACGT
T.urartu (EF081030)     CGGGAAGAAGGTGTTAGCTGGTTCTAAGAGAAAGGCGTTGGAGCTCGGCGG GGAGGACA     CGCCTCTCCCCAAGGAGGACG             GCC       CGAGCAAC   ATCGT
                        295                                                                                                                         200

Z.mays Lc (M26227)      CACCGTCACCGTCTCGGACAAGGACGTGCTCCTGGAGGTGCAGTGCCGGTGGGAGGAGCTCCTGATGACGCGAGTGTTCGACGCCATCAAGAGCCTCCATTTGGACGTCCTCTCGGTTCAGGCTT
O.sativa Ra (U39860)    CACCATCATGGACA   ACAAGGAGGTTCTCCTCGAGCTGCAATGCCAGTGGAAGGAATTGCTGATGACGAGAGTGTTCGACGCGATCAAGGGAGTCTCCCTGGATGTCCTCTCGGTGCAGGCAT
T.urartu (EF081030)     CAATGTCACCGTGACGGATAAAGAGGTGCTCCTTGAGGTGCAATGCCGGTGGAAGGAGCTACTGATGACACGAGTGTTCGACGCCATCAAGAGCCTCCGCCTGGACGTGTTGTCCGTCCGCGCGT
                        199                                                                                                                          75

                                                                i7
Z.mays Lc (M26227)      CAGCGCCAGATGGCTTCATGGGGCTTAAGATACGAGCTCAGTTTGCTGGCTCCGGTGCCGTCGTGCCCTGGATGATCAGCGAGGCTCTTCGCAAAGCTATAGGGAAGCGGTGA
O.sativa Ra (U39860)    CAACATCGGATGGTCTCCTTGGACTGAAGATACAGGCCAAGTTTGCCTCATCTGCTGCCGTCGAACCTGGGATGATTACAGAAGCTCTCCGGAAAGCTATAGCAAGCTAG
T.urartu (EF081030)     CCACGCCTGACGGCCTCCTCGCTCTCAAGATACGAGCTCAG
                        74                                       34
                                  1RP
```

Figure S2. *TaMyc1* structure and the placement of the overlapping amplicons used to derive the gene's full sequence. Primers used for 5′- and 3′-RACE shown in red.

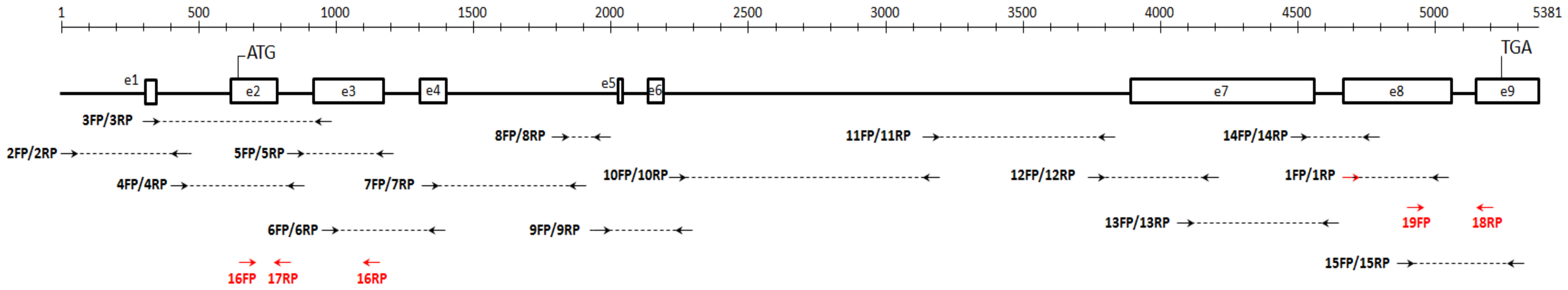

Figure S3. Alignment of wheat *Myc*-like sequences obtained by amplification with primer pair 1 (Table S2).

```
TaMyc1       TGAGACGATAACCTATCTCAGAGAGCTCGAACAGAAGGTGGAGGAGCTAGGATCCAACAGGGCGGCCAGAACAACAGCTG
TaMyc2       TGAGACGATAGCATATCTCAGAGAGCTGGAACAGAAGGTGGAGGAGCTAGAATCTAACAAGGCTGCCGGAACAACAGTTG
TaMyc3       TGAAACGATAGCCTACCTCAGAGAGCTGGAGCAGAAGGTGGAGGAGCTAGAATCCAACAGGGCGGCCG   CAACAGCCG
TaMyc4       TGAAACGATAGCCTACCTCAGAGAGCTGGAGCAGAAGGTGGAGGAGCTAGAATCCAACAGGGTGGCCG   GAACAGCCG
CONSENSUS    TGAGACGATAGCCTATCTCAGAGAGCTGGAACAGAAGGTGGAGGAGCTAGAATCCAACAGGGCGGCCGGAACAACAGCTG
             *** ****** * ** *********** ** ******************* *** **** **  ***     *****  *

TaMyc1       TCAGAAAACGCCATGAAGTCGGCGGGAAGAAGGTGTTAGCTCGTTCTAAGAGAAAGGCATCAGAGCTCGGCGGGGACGAC
TaMyc2       TCAGGAAACGTCATGAAGTCGGCGGGAAGAAGGTGTTAGCTGGTTCTAAGAGAAAGGCGTCGGAGCTCGGCGGGGAGGAC
TaMyc3       TCCGAAAACTCCATGACGTCAGCGGGAAGAAGGTGTTAGCTGGTTCCAAGAGAAAGGCGTCGGAGCTCGGCGGGGATGAC
TaMyc4       TCCGAAAACTCCATGACGTCAGTGGGAAGAAGGTGTTAGCTGGTTCCAAGAGAAAGGCGTCGGAGCTCGGCGGGGACGAC
CONSENSUS    TCAGAAAACGCCATGAAGTCGGCGGGAAGAAGGTGTTAGCTGGTTCTAAGAGAAAGGCGTCGGAGCTCGGCGGGGA.GAC
             ** * ****  ***** *** * ****************** **** *********** ** ************** ***

TaMyc1       ACGGAGAGGGTGCTCCCCAAGGACGACGGCCTGAGCAGTGTCATCAACGTCACCGTGACTGACAACGAGGTGCTCCTGGA
TaMyc2       ACGCCT      CTCCCCAAGGA   CGGCCCGAGCAACATCGTCAATGTCACCGTGACGGACAAAGAGGTGCTCCTTGA
TaMyc3       GCGGACAGGGTGCTCTCCAAGGAGGACGTCCCGAGCAACGTCGTCAACGTCACCGTGACGGAAAAAGAGGTGCTCCTGGA
TaMyc4       ACGGAGAGGGTGCTCCCCAAGGACGACGGCCCGAGCAACATCGTCAACGTCACCGTGACGGACAAAGAGGTGCTCCTGGA
CONSENSUS    ACGGA.AGGGTGCTCCCCAAGGA.GACGGCCCGAGCAACATCGTCAACGTCACCGTGACGGACAAAGAGGTGCTCCTGGA
              **         *** *******   ** ** *****   ** **** *********** ** ** *********** **

TaMyc1       GGTGCAATGCCGGTGGAAGGAGCTGCTGATGACACAAGTGTTCGACGCCATCAAGAGCCTCCGCCTGGACGTGCTCTCCG
TaMyc2       GGTGCAATGCCGGTGGAAGGAGCTACTGATGACACAAGTGTTCGACGCCATCAAGAGCCTCCGCCTGGACGTGTTGTCCG
TaMyc3       GGTGCAATGCCGGTGGAAGGAGCTGCTCATGACACAAGTGTTCGACGCCATCAAGAGCCTCCGCCTGGACGTGCTCTCCG
TaMyc4       GGTGCAATGCCGGTGGAAGGAGCTGCTGATGACGCAAGTGTTCGACGCCATCAAGAGCCTCCGCCTGGACGTGCTCTCCG
CONSENSUS    GGTGCAATGCCGGTGGAAGGAGCTGCTGATGACACAAGTGTTCGACGCCATCAAGAGCCTCCGCCTGGACGTGCTCTCCG
             ************************ ** ***** *************************************** * ****

TaMyc1       TGCGCGCGTCGACGCCCGA
TaMyc2       TCCGCGCGTCCACGCCTGA
TaMyc3       TGCGCGCGTCCACGCCCGA
TaMyc4       TGCGCGCGTCGACGCCCGA
CONSENSUS    TGCGCGCGTCCACGCCCGA
             * ******** ***** **
```

Figure S4. Physical mapping of the *TaMyc1-4* gene copies using "Chinese Spring" (CS) nulli-tetrasomic (**a**) and ditelosomic and deletion lines (**b**).

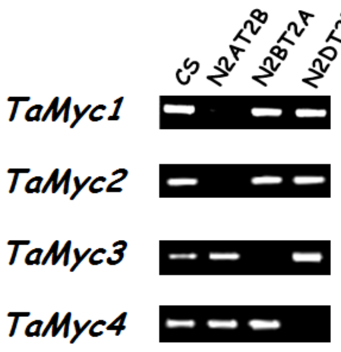

(**a**)

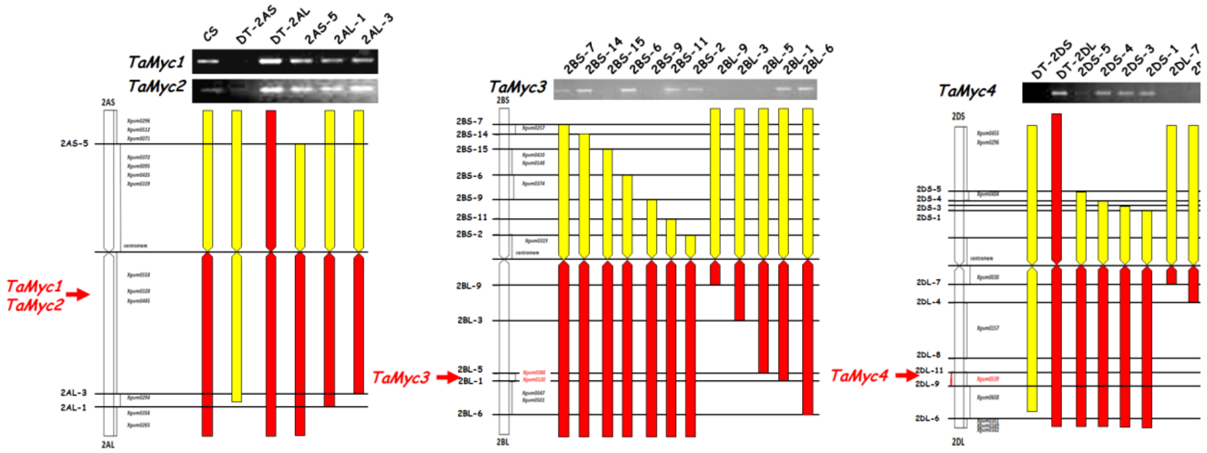

(**b**)

Figure S5. Alignment of maize, rice, wheat, barley, and *Arabidopsis* proteins participating in anthocyanin synthesis.

```
Wheat TaMYC1 (KJ747954)       MALPVVRPCQEEPTLPPPTGTQF SNQLAAAVRSINWSYAIFWSIS TSRPGVLTWKDGFYNGEIKTRKV
Rice RA (U39860)                         MEETPLPSGKNF RSQLAAAARSINWTYAIFWSIS TSRPGVLTWKDGFYNGEIKTRKI
Maize B (X57276)              MALSASPAQ  EELLQ PAGRPL RKQLAAAARSINWSYALFWSISSTQRPRVLTWTDGFYNGEVKTRKI
Maize LC (M26227)             MALSASRVQQAEELLQRPAERQLMRSQLAAAARSINWSYALFWSISDTQ PGVLTWTDGFYNGEVKTRKI
Barley ANT2(HM370319)          MALPIVRPSQEEP    PTGKQF SYQLAAAVRSINWSYAIFWSIS TSRPGVLTWKDGFYNGEIKTRKV
Arabidopsis TT8 (AJ277509)        MDESSIIPAEKVAGAEKKELQGLLKTAVQSVDWTYSVFWQFCPQQR  VLVWGNGYYNGAIKTRKT

Wheat TaMYC1 (KJ747954)       TSSADITADQLVLQRSEQLRELYESLLSGQCDHRAR  RPAAALSPEDLGDAEWYYTVCMGYAFRPGQGL
Rice RA (U39860)              TNSMNLTADELVLQRSEQLRELYDSLLSGECGHRAR  RPVAALLPEDLGDTEWYYVVCMTYAFGPGQGL
Maize B (X57276)              SHSVELTADQLLMQRSEQLRELYEALRSGECDRRGA  RPVGSLSPEDLGDTEWYYVICMTYAFLPGQGL
Maize LC (M26227)             SNSVELTSDQLVMQRSDQLRELYEALLSGEGDRRAAPARPAGSLSPEDLGDTEWYYVVSMTYAFRPGQGL
Barley ANT2(HM370319)          TSSADLTADQLLLQRSEQLRELYQSLLSGQCDHRGR  RPAAALSPEDLGDAEWYYAVCMSYAFRPGQGL
Arabidopsis TT8 (AJ277509)    TQPAEVTAEEAALERSQQLRELYETLLAGESTSEA   RACTALSPEDLTETEWFYLMCVSFSFPPPSGM

Wheat TaMYC1 (KJ747954)       PGRSFASNEHVWLCNAQCADTKTFQRALLAKTASIQTVACIP LMGGVLELGTTNTVLEDKGMVNWIGTS
Rice RA (U39860)              PGKSFASNEFVWLTNAQSADRKLFHRALIAKSASIKTIVCVPFIMHGVLELGTTDPISEDPALVDRIAAS
Maize B (X57276)              PGRSSASNEHVWLCNAHLAGSKDFPRALLAKSASIQTIVCIP LMGGVLELGTTDKVPEDPDLVSRATVA
Maize LC (M26227)             PGRSFASDEHVWLCNAHLAGSKAFPRALLAKSASIQSILCIP VMGGVLELGTTDTVPEAPDLVSRATAA
Barley ANT2(HM370319)          PGRSFASNEPVWLCNAQCADTKTFQRSLLAKTTSIQTVACIP LMGGVLELGTTDTVLEDRDMVNRISTS
Arabidopsis TT8 (AJ277509)    PGKAYARRKHVWLSGANEVDSKTFSRAILAKSAKIQTVVCIP MLDGVVELGTTKKVREDVEFVELTKSF

Wheat TaMYC1 (KJ747954)       FWELKFPTCSKSEEPNSIPSVDDTGDADIVFDVLNHNTM    AMMIPG    ELELGEVECLS   DDN
Rice RA (U39860)              FWDTP         PRAAFSSEAGDADIVVFEDLDHGNAAVEATTTTVPGEPHAVAGGEVAECEPNADND
Maize B (X57276)              FWEPQCPTYSKEPSSNPSAYETGEAAYIVVLEDLDHNAMDMETVTAAAGRHGTGQELGEVESPS   NAS
Maize LC (M26227)             FWEPQCPSSSPSGRANETGEAAADDGTFAFEELDHNNGMDDIEAMTAAGGHGQEEELRLREAEALSDDAS
Barley ANT2(HM370319)          FWDLKIPTSSKPKEPSS PSADDAGEADIVFQDLDHNTM    AAMIPG    ELELGEVECLS   DDN
Arabidopsis TT8 (AJ277509)    FYDHC        KTNPKPALSEHSTYEVHEEAEDEEEVEEEMTMSE      EMRLGSPDDEDVSNQNL

Wheat TaMYC1 (KJ747954)       LERITKE INRFYGLYDELDVGA    LEENWTMGG  C FE IMSSPEVP   PAPAATNGITNGAVTL
Rice RA (U39860)              LEQITMDDIGELYSLCEELDVVRPLDDDSSSWAVADPWSSFQLVLTSSPAPDQAPAAEATDVDDVVVAAL
Maize B (X57276)              LEHITKG IDEFYSLCEEMDVQ    PLEDAWIMDG  SNFE VPSSA                 LPVDG
Maize LC (M26227)             LEHITKE IEEFYSLCDEMDLQALPLPLEDGWTVDA  SNFE VPCSSPQP   APPPVDRATANVAADA
Barley ANT2(HM370319)          LERITKE INGFYGLCDELDVGA    LDENWIIGG  S FE VMSSPEAP   PAPAATGGITDGIVTL
Arabidopsis TT8 (AJ277509)    HSDLH   IESTHTLDTHMDMMNL   MEEGGNYS   QTVTTLLMSHP          TSLLSDSVSTY

Wheat TaMYC1 (KJ747954)       SSVEP  S   RSSCFTAWKRSWD  SAEDMAT     LVARETQKLLKKALAGG AWANNGGDD  TAR
Rice RA (U39860)              DGSSIDGSCRPSPSSFVAWKRTAD  SDEVQAVP   LISGEPPQKLLKKAVAGAGAWMNNG DSSAAAM
Maize B (X57276)              SSAPADGS   RATSFVVWTRSSH  SCSGEAA    VPVIEEPQKLLKKALAGGGAWANTNCGGGGTTV
Maize LC (M26227)             SRAPVYGS   RATSFMAWTRSSQQSSCSDDAAPAAVVPAIEEPQRLLKKVVAGGGAWESCG GATGAAQ
Barley ANT2(HM370319)          SAAAS  S   LSSCFTAWKRSWD  SAEDMAA     PVAGQSQKLLKKALAGG VWAINGGGGGGTAR
Arabidopsis TT8 (AJ277509)    SYIQ         SSFATWRVENGKEHQQVKTAP        SSQWVLKQMIFRVPFLHDN      TKD

Wheat TaMYC1 (KJ747954)       AQESTNTKNHVISERRRREKLNEMFLILKSLVPSIHKVDKASILAETITYLRELEQKVEELGSNRAART
Rice RA (U39860)              TTQESSIKNHVMSERRRREKLNEMFLILKSVVPSIHKVDKASILAETIAYLKELEKRVEELESSSQPSPC
Maize B (X57276)              TAQENGAKNHVMSERKRREKLNEMFLVLKSLVPSIHKVDKASILAETIAYLKELQRRVQELESRRQ
Maize LC (M26227)             EMSGTGTKNHVMSERKRREKLNEMFLVLKSLLPSIHRVNKASILAETIAYLKELQRRVQELESSREPASR
Barley ANT2(HM370319)          AQESSNTKNHVISERRRREKLNEMFLILKSLVPSIHKVDKASILAETIAYLRELEQRVEELESNRAPSRP
Arabidopsis TT8 (AJ277509)    KRLPREDLSHVVAERRRREKLNEKFITLRSMVPFVTKMDKVSILGDTIAYVNHLRKRVHELENT

Wheat TaMYC1 (KJ747954)         TAV        RKRHEVGGKKVLARS  KRKASELGGDDTER  VLPKDDGLSSVINVTVTDNEVLLE
Rice RA (U39860)              PLETRSR      RKCREITGKKVSAGAKRKAPAPEVASDDDTDG   ERRHCVSNVNVTIMDNKEVLLE
Maize B (X57276)                            GGSGCVSKKVCVGSNSKRKSPEFAGGAKEHPWVLPMD GTSNV TVTVSDTNVLLE
Maize LC (M26227)             PSETTTRLITRPSRGNNESVRKEVCAGS  KRKSPELGRDDVERPPVLTMDAGTSNV TVTVSDKDVLLE
Barley ANT2(HM370319)          AGAAV        RRHHDAAAKKMLAGS  KRKASELGGDD           GPNSVVNVTVTEKEVLLE
Arabidopsis TT8 (AJ277509)                   HHEQQHKRTRTC   KRKTSE                    EVEVSIIENDVLLE

Wheat TaMYC1 (KJ747954)       VQCRWKELLMTQVFDAIKSLRLDVLSVRASTPDDLLALKIRAQFAGPGVVETGMISEALQRAIRRP
Rice RA (U39860)              LQCQWKELLMTRVFDAIKGVSLDVLSVQASTSDGLLGLKIQAKFASSAAVEPGMITEALRKAIAS
Maize B (X57276)              VQCRWEKLLMTRVFDAIKSLHLDALSVQASAPDGFMRLKIGAQFAGSGAVVPGMISQSLRKAIGKR
Maize LC (M26227)             VQCRWEELLMTRVFDAIKSLHLDVLSVQASAPDGFMGLKIRAQFAGSGAVVPWMISEALRKAIGKR
Barley ANT2(HM370319)          VQCRWKELLMTQVFDAIKSLRLDVLSVRASTPDGLLALKIRAQFAGPGAVEPGMIIGALQTATRGR
Arabidopsis TT8 (AJ277509)    MRCEYRDGLLLDILQVLHELGIETTAVHTSVNDHDFEAEIRAKVRGKKASIAEVKRAIHQVIIHDTNL
```
